# Supplementary figures and images for: Lake Ecosystem Robustness and Resilience Inferred from a Climate-Stressed Protistan Plankton Network
Source: Microorganisms. 2021 Mar 6;9(3):549. doi: 10.3390/microorganisms9030549 (PMC8001626; doi:10.3390/microorganisms9030549)

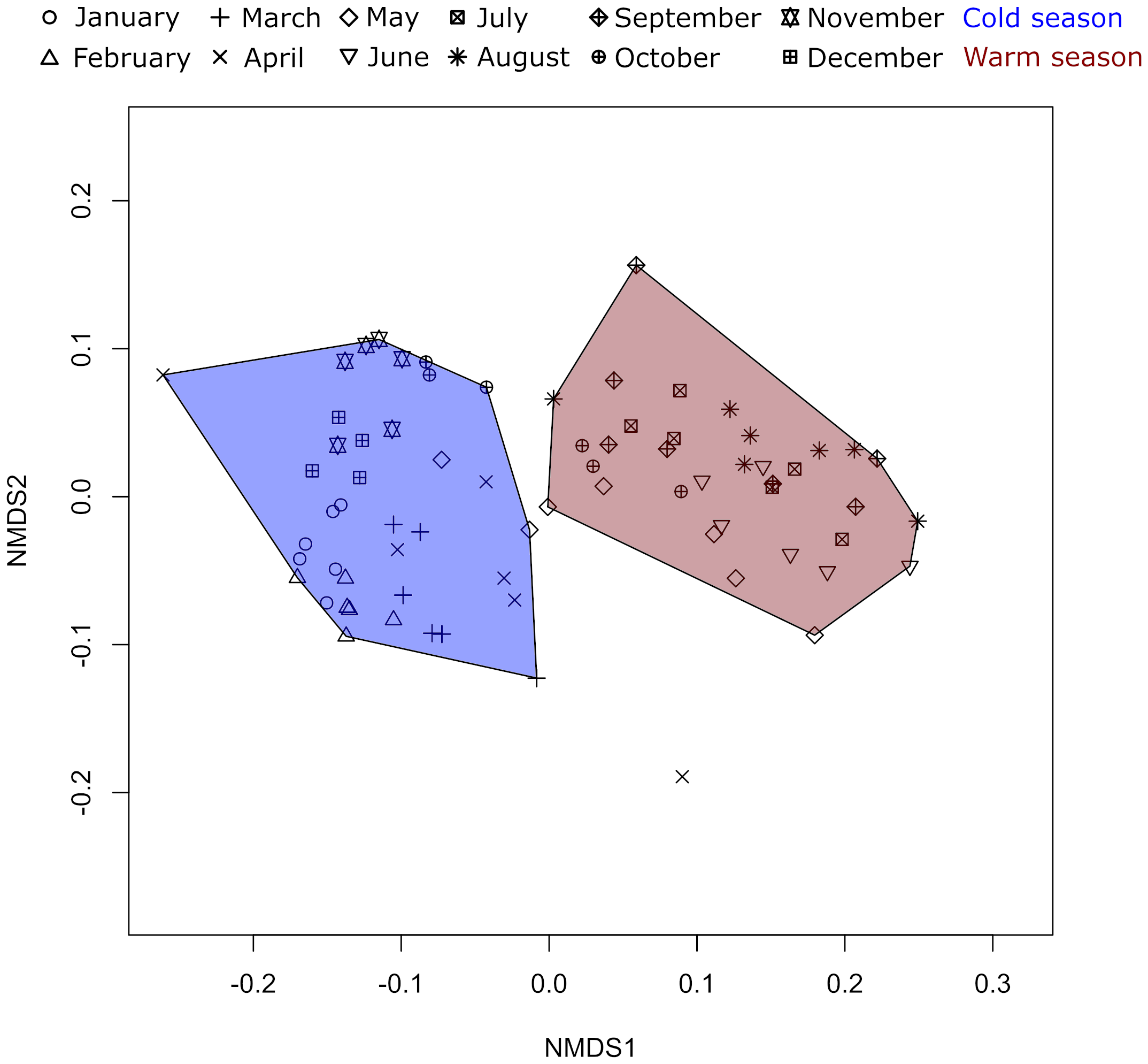

Supplement: Supplementary file 1 [file microorganisms-09-00549-s001.zip › Figure_S1.tiff]
